# Nash Equilibria in Multi-Agent Motor Interactions

**DOI:** 10.1371/journal.pcbi.1000468

**Published:** 2009-08-14

**Authors:** Daniel A. Braun, Pedro A. Ortega, Daniel M. Wolpert

**Affiliations:** 1 Computational and Biological Learning Laboratory, Department of Engineering, University of Cambridge, Cambridge, United Kingdom; 2 Bernstein Center for Computational Neuroscience, Freiburg, Germany; University College London, United Kingdom

## Abstract

Social interactions in classic cognitive games like the ultimatum game or the prisoner's dilemma typically lead to Nash equilibria when multiple competitive decision makers with perfect knowledge select optimal strategies. However, in evolutionary game theory it has been shown that Nash equilibria can also arise as attractors in dynamical systems that can describe, for example, the population dynamics of microorganisms. Similar to such evolutionary dynamics, we find that Nash equilibria arise naturally in motor interactions in which players vie for control and try to minimize effort. When confronted with sensorimotor interaction tasks that correspond to the classical prisoner's dilemma and the rope-pulling game, two-player motor interactions led predominantly to Nash solutions. In contrast, when a single player took both roles, playing the sensorimotor game bimanually, cooperative solutions were found. Our methodology opens up a new avenue for the study of human motor interactions within a game theoretic framework, suggesting that the coupling of motor systems can lead to game theoretic solutions.

## Author Summary

Human motor interactions range from adversarial activities like judo and arm wrestling to more cooperative activities like tandem riding and tango dancing. In this study, we design a new methodology to study human sensorimotor interactions quantitatively based on game theory. We develop two motor tasks based on the prisoner's dilemma and the rope-pulling game in which we introduce an intrinsic cost related to effort rather than the typical monetary outcome used in cognitive game theory. We find that continuous motor interactions converged to game theoretic outcomes similar to the interaction dynamics reported for other dynamical systems in biology ranging in scale from microorganisms to population dynamics.

## Introduction

Riding a tandem, tango dancing, arm wrestling and judo are diverse but familiar examples of two-player motor interactions. The characteristic feature of such interactions is that the two players influence each others behavior through coupled sensorimotor control with continuous action spaces over repeated trials or continuously in time. In contrast, two-player interactions considered in classical game theory are typically thought to involve cognition in games with discrete actions and discrete time steps for decision-making such as tic-tac-toe, the ultimatum game or the prisoner's dilemma [1]–[8]. An important concept in such classical games is the *Nash equilibrium solution* [9] in which each player chooses a strategy such that no player has anything to gain by changing only his or her strategy. Nash equilibria can also be defined for continuous games, i.e. games with continuous actions and payoffs [10]–[12], and thus might provide a theoretical tool to understand multi-agent sensorimotor interactions. The theory of continuous games can also be used for sequential (dynamic) games where players are interacting continuously over a sequence of time steps [13]–[15]. Nash equilibria in such continuous dynamic motor games correspond to (equilibrium) control policies, i.e. feedback rules that map past observations to actions.

**Figure 1:**
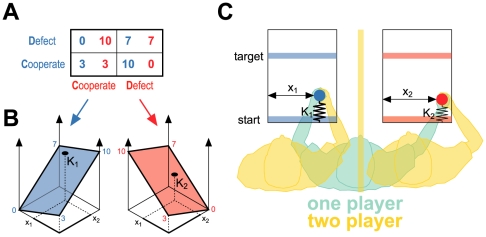
The prisoner's dilemma motor game. (A) Pay-off matrix for the classical prisoner's dilemma for two players (players denoted by red and blue). Depending on the choice of each player there are four different outcomes in terms of years that each player will serve in prison. (B) The motor version of the prisoner's dilemma. Each player controls a cursor and moves from a starting bar to a target bar and experiences a force that resists forward motion. The force arises from a virtual spring that attaches the handle to the starting bar (the springs are only shown on the schematic and are not visible to the players). The stiffness of the springs (K_1_ & K_2_) can vary online and each depends on the x-positions of both players' cursors (x_1_ & x_2_). (C) Continuous cost landscape for the motor prisoner's dilemma game. Each pair of x-positions (x_1_, x_2_) corresponds to a spring constant for each player. The corners of the plane correspond to the classical prisoner's dilemma matrix (A) and intermediate spring constants are obtained by linear interpolation. The current spring constants experienced by the players in B are shown by the points on the surface. The game was played by eight pairs of players and by eight individual players bimanually.

Here, we develop continuous sensorimotor versions of the prisoner's dilemma and the rope-pulling game. In the classical prisoner's dilemma [16], two players (prisoners) have a choice (Fig. 1A) between cooperation (claiming the other player is innocent) and defection (claiming the other player is guilty). If both cooperate, they each receive a short sentence (3 years) whereas if both defect they each receive a moderate sentence (7 years). But if one cooperates while the other defects, the defector is freed and the cooperator receives a lengthy sentence (10 years). The globally optimal solution in which the players benefit the most is for both players to cooperate. However, if one of the players decides to defect, the defector reduces their sentence at the expense of the other player. In such a non-cooperative setting the stable Nash solution is for both players to defect. This Nash solution guarantees in this case that a player minimizes their maximum expected punishment (in this case 7 years) and the player does not have to rely on a particular action being chosen by the other player. The dilemma arises because the Nash solution is not identical to the globally optimal solution which is cooperative. The same dilemma occurs also in the rope-pulling game (given as a conceptual example in [15]) where each of two players is attached by a rope to a mass that they have to pull together. One player is rewarded according to how far he pulls the mass along one direction and the other player is reward according to how far he pulls the mass in an orthogonal direction. Thus, the globally optimal solution is to cooperate and pull the mass along the diagonal. However, if one of the players defects and pulls into his own direction he gains even more payoff at the expense of the other player. Therefore, the stable Nash solution in this case is for each player to pull along his own direction. In the following we address the question whether human motor interactions in such motor games can be quantified using a game theoretic framework.

## Results

In our continuous sensorimotor version of prisoner's dilemma (see Materials and Methods), two players sat next to each other and grasped the handles of separate robotic interfaces that were free to move in the horizontal plane (Fig. 1B, orange players). A virtual reality system was used to overlay visual feedback onto the plane of movement and players were prevented from seeing their own hand or that of the other player [17]. Each player controlled the position of a cursor that represented the position of their hand. On each trial, the players were required to move their cursors to touch target bars which were directly ahead of them. However, participants were free to move the handle laterally to touch the target bar anywhere along its width. Therefore, participants could achieve the task with their final hand position anywhere between the left and right target bounds. One bound (e.g. left) represented cooperation while the other bound (e.g. right) represented defection. An implicit pay-off was placed on the movements by using each robot to generate a resistive force opposing the forward motion of the handle. The forces were generated by simulating springs that acted between each handle and its starting bar. The stiffness of each spring could vary continuously during the movement depending on the lateral positions of both handles. Directly analogous to the prisoner's dilemma, the spring constants depended on whether the two players cooperated or defected. That is we translate the sentence in years in the traditional cognitive game (Fig. 1A) into spring constants in N/m in our sensorimotor game. For positions of the handles between the bounds, that is between full cooperation and full defection, we linearly interpolated these spring constants (Fig. 1C shows spring constants landscape for each player). Therefore, the actions of each player directly affected both the forces they experienced, as well as the forces experienced by the other player. The game was either played by two players (Fig. 1B, orange) or by one player bimanually (Fig. 1B, green). We hypothesized that the bimanual condition could be conceived of as two cooperating players (instantiated by the two brain hemispheres) which should result in cooperative solutions as opposed to the competitive Nash solutions expected for the two-player setup. Each session consisted of 20 sets with each set consisting of 40 trials. At the start of each set the assignment of the defect/cooperation boundaries to the left/right side of each target was randomized.

**Figure 2:**
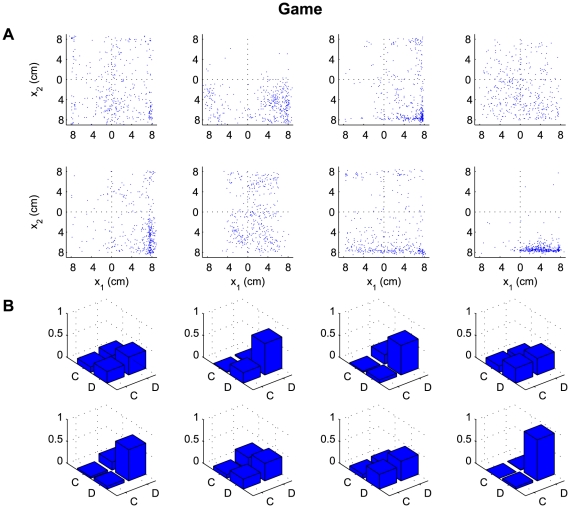
Results of the two-player version of the prisoner's dilemma. (A) Endpoint distribution of handle positions in the four quadrants corresponding to the cooperate defect (lateral movement) plane with the cooperative solution (top left quadrant), the Nash solution (bottom right quadrant) and the two exploitative solutions (top right or bottom left quadrant). Each plot shows one of the eight games in the two-player version of the prisoner's dilemma. The data is shown for the last 20 trials in each set. (B) Histogram over the four quadrants. C corresponds to cooperation and D to defection. All eight participant-pairs show a strong tendency towards the Nash solution.

**Figure 3:**
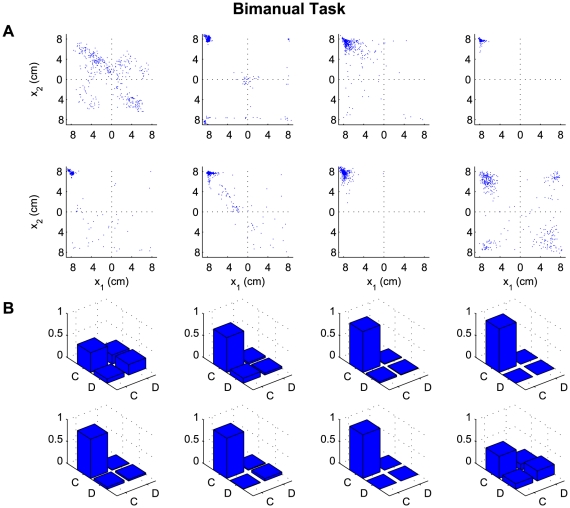
Results of the one-player bimanual version of the prisoner's dilemma. (A) Endpoint distribution of handle positions in the four quadrants corresponding to the cooperate defect (lateral movement) plane with the cooperative solution (top left quadrant), the Nash solution (bottom right quadrant) and the two exploitative solutions (top right or bottom left quadrant). Each plot shows one of the eight participants. The data is shown for the last 20 trials in each set. (B) Histogram over the four quadrants. C corresponds to cooperation and D to defection. All eight participants had a strong preference for the cooperative solution.

Thus, our motor version of the prisoner's dilemma differs from the classic discrete version of the game in at least three different aspects. First, actions are continuous such that there is a continuous coupling between the two players. Second, reward in terms of money or years is replaced by an implicit cost, that is effort. Third, subjects have to learn their optimal strategy since they are unaware of the structure of the coupling, i.e. they have incomplete information about the payoffs. We found a clear distinction between the strategies used at the end of a set for the one-player and the two-player conditions. In Figure 2A and Figure 3A we show the endpoint distributions of the action choices for the two-player and the bimanual conditions. To analyze this result we categorized the final positions of the cursors in Figure 2B and Figure 3B into defect and cooperate responses, that is Nash responses (defect-defect), cooperative responses (cooperate-cooperate) and exploitative responses (defect-cooperate or cooperate-defect). In the one-player condition, the globally optimal cooperative solution was chosen in the majority of instances (Fig. 3B). In contrast, although in some of the two-player games there was a small fraction of exploitative trials, the two-player game led mostly to the Nash solution (Fig. 2B). The globally optimal cooperative solution was seen significantly more often for the one-player game compared to the two-player game (p<0.01, Wilcoxon ranksum test on number of cooperative solutions in the two-player versus the bimanual condition) and conversely the Nash solution was seen significantly more often (p<0.01) for the two-player game than for the one-player game.

**Figure 4:**
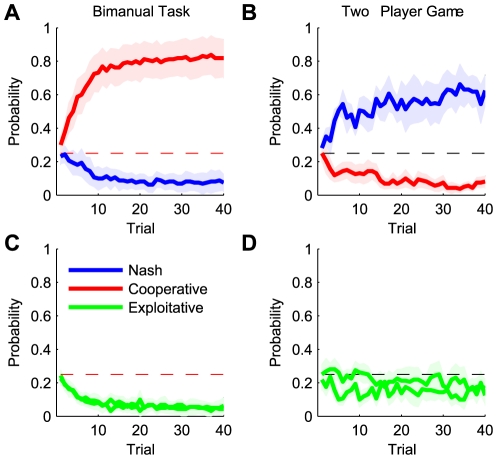
Temporal evolution of game solutions. (A,B) The evolution of the probability of cooperative (blue) and Nash (red) solutions across a 40 trial set for the one-player and two-player conditions. In the one-player condition the cooperative solution gains most probability, where as in the two-player condition the Nash solution is predominant. (C,D) The evolution of the probability of the exploitative solutions across the same set of trials for the one-player and two-player conditions. The shading is one standard error of the mean across the participants. As there are four possible behaviors chance level is shown at a probability of 0.25.

To investigate the temporal evolution of learning we analyzed the trial-by-trial behavior of the players averaged across all sets. Initially, in both the one-player and the two-player conditions, players acted at chance level in their strategy (Fig. 4). Later trials of the one-player game converged to the globally optimal cooperative solution, while their probability of choosing a Nash solution dropped to close to zero (Fig. 4A). In contrast, players in the two-player condition showed an increasing tendency to act according to the Nash solution over the course of a set, while their probability of choosing a cooperative solution dropped significantly below chance level (Fig. 4B). The frequency of the exploitative solutions decreased in the bimanual condition along with the frequency of the Nash solution (Fig. 4C). In the two-player game on the other hand, the frequency of exploitative solutions stayed around chance level (Fig. 4D). Therefore, players in both conditions showed significant exploration and learning over trials.

**Figure 5:**
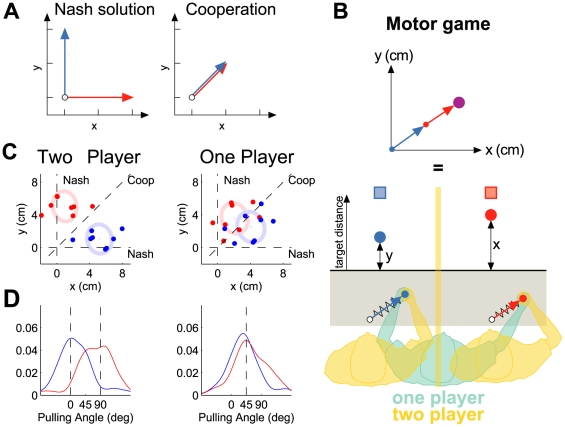
The rope-pulling game. (A) The rope-pulling game in which a mass (circle) is pulled by two players. The arrows show the direction of force for two players for the Nash and cooperative solutions. Red and blue colors represent right and left handles throughout. (B) The motor version of the rope-pulling game. The position of a virtual mass is the sum of the displacements of the two handle positions from their origin (blue and red displacement vectors). However, the visual feedback is only a one-dimensional cursor location that is the y and x values of the mass position for players 1 and 2 respectively. Each player is required to reach a visual target with their cursor. Each robot was used to simulate the forces that would arise from a spring (with constant stiffness) attached between the handle and its origin. The arrow vectors and springs are only shown on the schematic and are not visible to the participants (grayed area not visible). For the one player game, a single participant controls both handles. The game was played by 4 pairs of participants and by 4 different participants individually. (C) Mean end points for each pair of left and right players for the last 40 trials in each set. The ellipses are centered at the average end points across all participants and indicate one standard error. (D) Smoothed frequency histograms (Gaussian kernel sd 20°) of pulling angles for the two-player condition (left: Nash equilibrium shown by vertical lines) and for the one player condition (right: cooperative solution shown by vertical line).

In our sensorimotor version of the prisoner's dilemma the cooperative and Nash solutions are two extremes of the one-dimensional control variable (lateral position at the target bar). Therefore, we designed a motor task based on another game, the ‘rope-pulling-game’, which has three additional features. First, the control variable is two-dimensional and the Nash and cooperative solutions are no longer at the boundaries of the control space. Second, unlike the prisoner's dilemma, where each player can achieve their task (reaching the bar) without paying attention to the strategy of the other player, in the new task, coordination is required between the players to jointly achieve the task. Third, the rope-pulling game can be translated into a linear dynamical system allowing for analytical solutions in terms of feedback policies (see Text S1 for details). In the rope-pulling-game, two players each pull on a rope attached to a mass (Fig. 5A). Player 1 and player 2 are rewarded according to how far each manages to pull the mass along the y- and x-axis respectively. If the players cooperate they should both pull along the diagonal (Fig. 5A, right), because in this way no forces are wasted compensating for the other player's force. However, if one of the players decides to defect and pull only in his own direction while the other pulls along the diagonal, the defector increases his reward at the expense of the other player. In such a non-cooperative setting the stable Nash solution is for each player to pull only in his own direction (Fig. 5A, left).

In our version of the rope-pulling game, player 1 and 2's task was to move a virtual mass to a fixed target that was equidistant to both players' origin. Again, each participant grasped the handle of the robotic interface and the location of the virtual mass was the sum of the (possibly rotated—see Materials and Methods) positional displacements of the two handles from their origin (Fig. 5B, red and blue arrow vectors) – just like the positional vectors of two real agents would add up when pulling a real mass. Accordingly, there are infinitely many solutions to reach the target as there are infinitely many ways of how to add two vectors to become a certain target vector. However, each player only saw a one-dimensional projection of the two-dimensional position of the virtual mass, i.e. one player saw a projection along the virtual x-axis and the other player saw a projection along the virtual y-axis corresponding to the direction that they had to control (Fig. 5B). An implicit pay-off was placed on the movements by using the robots to simulate stiff springs between each handle and its origin. Therefore, the further a player had to move the robotic handle from the origin to achieve the task, the greater the resistive force and hence effort required. Limiting each participant's visual feedback to one-dimension, while preventing them from seeing the other participant's feedback, ensures that they could not form an explicit representation that could be used for cognitive solutions. In contrast to the prisoner's dilemma task the actions of each player did not directly affect the forces experienced by the other player but directly affected the position of the other player's cursor. Therefore, each cursor was affected by both players requiring coordination between the players to achieve the task, because subjects could not disregard the other player's action choice as both actions had to add up to a fixed target value. The game was either played by two players (Fig. 5B, orange) or by one player bimanually (Fig. 5B, green) as in the previous game.

We analyzed the distribution of pulling directions after learning (Fig. 5C, positions & 5D angles). We found that when a single participant played the game bimanually the movements tended to converge to the cooperative solution with pulling directions clustered around 45° for both arms. In contrast, in the two-player game the directions tended to converge to the Nash solution with pulling directions around 0° and 90° for the two participants. In individual games, both single players and pairs of players could deviate substantially from their respective solutions. Importantly, however, the densities of the solutions in the one-player and two-player conditions were different. Examining the average solution for each participant in the two player game and for each arm in the one-player game showed that the two-player game deviated significantly from 45° (p<0.01, Wilcoxon signrank test), while the one-player game did not (p>0.1). Thus, individual players tended towards a cooperative solution between the two arms, whereas two players tended towards a Nash equilibrium on average.

## Discussion

In our study we have assessed human sensorimotor interactions based on game theoretic predictions with an implicit cost, that is effort. Effort, as a proxy for energy consumption, has been shown to be a fundamental determinant underlying how humans control their own movements [18],[19]. In line with the game theoretic predictions, we found in both motor games that the Nash equilibrium was the predominant solution in two-player motor interactions and the cooperative solution dominated in the one-player interactions. Previous studies have shown that natural patterns of coordination can arise between participants when provided with feedback of the other participant, such as the synchronization of gait patterns (for a review see [20]). In distinction, in our study we limited knowledge of the other participants' behavior to a pay-off in terms of energy and showed that different patterns of interaction develop in the one-player and two-player conditions that can be explained within the game theoretic framework. While this is a different explanatory framework, one should bear in mind that optimality theories and dynamic systems theory are in principle compatible with each other [21]. In previous studies in psychology, human group behavior in physical tasks such as tug-of-war has been examined and compared to individual performance. It was found that individuals tend to reduce their effort in group tasks and instead rely on others, for example in force production in tug-of-war [22],[23]. This has been dubbed “social loafing”, but has not been examined in a game theoretic context. Yet, game theoretic analysis has been applied to a wide range of biological systems from interacting microorganisms [24],[25], through animal behavior [26],[27] to understanding population dynamics [28].

Our results contrast with those obtained in cognitive discrete games in interesting ways. For example, in the classical prisoner's dilemma, contrary to game-theoretic predictions, cooperation plays a significant role: players have been reported to cooperate almost half the time [29],[2]. Consequently, a large number of studies have investigated experimental and theoretical conditions that allow for such cooperation [30]–[34]. Especially, in iterated versions of the prisoner's dilemma it was found that cooperative strategies such as tit-for-tat or “win-stay lose-shift” can be very successful [35]–[37]. While cooperation can be optimal in case of indefinite repetitions [38], for a fixed number of iterations it is still optimal to defect.

In our motor version of the prisoner's dilemma the participants showed very little inclination towards cooperative solutions. This could have several reasons. Our participants knew, for example, that the experiment was going to last for 800 trials, i.e. assuming the participants had full knowledge of the game structure their defection is optimal – however, knowing the number of trials does not stop players in discrete cognitive games from cooperating. In our study the action space is continuous. A recent theoretical study has found, for example, that cooperative solutions are less stable in continuous environments where agents can make gradual distinctions of cooperativeness ranging from full cooperation to total defection [13]. The intuition behind this finding is that the deadlock of a non-cooperative equilibrium is more difficult to break by agents that cooperate only slightly more than their non-cooperative counterparts, because two marginally cooperating agents have much less to gain from each others cooperation than two tit-for-tat agents, for example, that try full cooperation. To investigate the impact of action continuity on human cooperativeness in games one could compare the outcomes of continuous prisoner's dilemma experiments with monetary feedback to the outcomes of discrete versions of the game. Another important difference of both our motor games compared to classic game theoretic settings is that players had incomplete information about the payoff function and the structure of the game. Thus, players first had to gather information and learn the structural determinants of the game. Again one could compare our results to the outcome of classical prisoner's dilemma games where participants are not informed about payoff functions. Furthermore, due to the motor nature of the interactions, psychological effects such as ‘mentalizing’ might have been reduced [39]. Participants in our motor games were not aware of the effects of their actions on the other player, since each player could not feel the force feedback given to the other player. To test whether such psychological effects would have an influence in our games that could lead towards more cooperation, one could give explicit feedback about the other player's payoff (e.g. force display in Newtons) and explain that their choice of action affects the other player's toil. Finally, it would also be interesting to investigate more complex games, since we have only examined a special class of games where the Nash solution corresponds to a minimax-solution – this is in general true for zero-sum games [40], and also for the prisoner's dilemma. For this solution type, a player minimizes the maximum expected loss, thereby ignoring the actions of the other player (both in the classical and the motor prisoner's dilemma game). Thus, more complex games with more complex cost functions provide an interesting avenue for future research.

In our second motor game, the rope-pulling game, over all players we still observed that the Nash solution was the predominant solution for two-player interactions, but this time the inter-subject differences were quite considerable both in the two-player condition and in the bimanual case. One reason for this could be that the task was substantially more complex than the prisoner's dilemma task, especially in the bimanual case where two two-dimensional movements had to be performed simultaneously. Thus, incomplete learning might have played a crucial role. To model such states of incomplete information a special theory of Bayesian games has been devised dealing with so-called Bayes-Nash solutions [41] that need not to correspond to Nash equilibria in the same game under complete information. Furthermore, the dynamics of learning in two-player interactions are also studied in the reinforcement learning literature from a single-agent perspective [42]–[46]. Here we restricted ourselves to simpler classic game-theoretic models to analyze two-player motor interactions assuming complete information, i.e. complete learning of the true payoff structure – the same assumption is typically made in other optimal control models where learning itself is not modeled [19],[18]. Indeed, cooperation in our game can be modeled using optimal feedback control theory [18] – in Text S1 we indicate how normative and methodical principles from optimal feedback control can be carried over to game theoretic settings. Although dynamic game theory is the most general formulation of motor games, it is not the only tool available to model sensorimotor interactions. For some games, such as those considered in our experiments, simple geometric considerations can be sufficient. In our games players also had imperfect information about the actions of the other player, i.e. they only felt the consequences of the other player's actions without feeling the force feedback given to the other player. This does not invalidate our model, however, since in our games players did not have to know the other players actions to play the Nash equilibrium policy, because, as already mentioned, Nash equilibria in our games corresponded to minimax-solutions. This is ultimately a consequence of the structure of the cost functions we employed, since each players payoff function did not take the actions of the other player into account explicitly. In the future it will be interesting, therefore, to investigate human motor interactions in games with more complex cost functions and to apply more advanced modelling tools.

In both our motor games we compared performance of two-players with the performance of a single player. The underlying hypothesis was that the single player condition could be regarded as an instance of a cooperative game where the two motor hemispheres interact to achieve the task. If the two hemispheres were unable to cooperate, for example as might be expected in patients who have undergone commisurectomy [47], then Nash equilibria might also arise in a single player. In summary, our results suggest that sensorimotor interactions can be understood by a game theoretic framework and that cooperative and Nash solutions in motor interactions can arise naturally by the dynamical coupling of two interacting sensorimotor processes. Moreover, the general design of our experiments provides a tool to translate classical games into continuous motor games and might provide a new avenue for studying human motor interactions.

## Materials and Methods

Forty-eight naïve participants provided written informed consent and took part in one of two motor games. The experiments were conducted using two planar robotic interfaces (vBOTs). Participants held the handle of the vBOT that constrained hand movements to the horizontal plane. The vBOT allowed us to record the position of the handle and to generate forces on the hand with a 1 kHz update rate. Using a projection system we overlaid virtual visual feedback into the plane of the movement [17].

### 

#### Ethics statement

All experimental procedures were approved by the Psychology Research Ethics Committee of the University of Cambridge.

#### Prisoner's dilemma motor game

Each of the robot handles controlled the position of a cursor in one half of the horizontal workspace (Fig. 1B). The cursor could be continuously controlled within a single trial. Each participant's task was to place their cursor within their respective target bar. A trial started after both participants had placed their cursor stationery within their respective starting bar. Both target bars then appeared at a distance randomly drawn each trial between 5 and 20 cm (the same distance for both players on each trial). Participants were required to make a forward movement (y-direction) to touch the target bar. They were free to touch it anywhere along its 15 cm width (the robot simulated walls which prevented participants moving further laterally than the width of the bar). For successful trial completion, the target bar had to be reached by both players within 1500 ms. The final *x*-position was taken as their choice in the game. During the movement, both players experienced a one-dimensional spring attached to the starting bar. The spring constants depended on the lateral positions 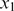 and 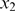 of both players, where 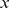 corresponds to a normalized lateral deviation ranging between 0 and 1. For each target bar, one edge was defined as defect (e.g. 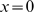) and the other as cooperate (e.g. 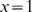). The assignment of the defect/cooperation boundaries to the left/right side of each target could be randomized. This gave four possible assignments (i.e. defect to left or right of target bar 1 and defect to left or right of target bar 2). Intermediate lateral deviations took on values between 0 and 1. The final *x*-position was categorized as cooperate or defect depending on whether 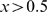 or 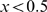. The spring constants were continuously updated as 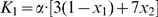 and 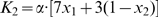, for players 1 and 2 respectively. The scaling parameter 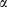 was constant throughout the experiment at 0.19 N/cm. These spring constants are linear interpolations of the classical prisoner's dilemma matrix (Fig. 1A), with intermediate lateral deviations leading to intermediate spring constants (Fig. 1C). The participants experienced forces 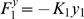 and 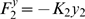 resisting their forward motion in which 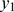 and 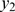 are the y-distances of player 1 and 2's hands from the starting bar respectively. Participants performed 20 sets of 40 trials. At the start of each set the allocation of the target edges to defect/cooperate was randomized. Thus, within a set of 40 trials the same force landscape was applied.

#### Rope-pulling game

The position of each robot handle was expressed as a two-dimensional vector position where 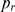 and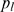 are the position of right and left robot handle, respectively. The two robot handle positions together determined the position of a virtual mass at 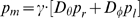 where 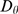 and 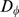 are 2×2 rotation matrices. The scaling parameter was set to 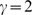 throughout the experiments in order to confine arm movements to a smaller workspace so as to avoid collision of the robot handles. The rotation matrices were introduced to factor out any preference of movement direction and to allow repetitions of the game with different solutions. The rotations for each robot, 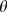 and 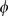, were drawn randomly from [−135°, −90°, −45°, 0°, +45°]. For successful trial completion, the virtual mass had to be placed on the virtual target at (13,13) cm for 200 ms within a time limit of 1500 ms. Therefore, participants had to move in two dimensions so as to place the one-dimensional cursor in the target. Accordingly, there are infinitely many solutions to reach the target as there are infinitely many ways of how to add the two position vectors to equal the target vector. However, neither the virtual mass point position nor the virtual target was displayed in 2-dimensional space. Participants could only see a one-dimensional projection of the virtual mass point such that player 1 saw the y-component of the position and player 2 saw the x-component of the virtual mass point position. This corresponded to the dimension that they had to control. Additionally, an isotropic spring (5 N/cm) was simulated attached from the handle of each robot to its origin. This increased the effort required for larger movements. Each game consisted of 10 sets of 80 trials with the same visuomotor rotations. Visual feedback was provided continuously throughout the movement. The final cursor position was taken as the players' choice in the game and used to compute the pulling angles. The feedback ensured that participants were never aware of playing a version of the rope-pulling game.

Each game was played by eight pairs of participants and by eight different participants individually. All participants were instructed to achieve the task as easily as possible. Participants were also told the number of trials in each set, and the sets were separated during the experiment by short breaks. For the two-player game a divider was used to prevent the participants seeing the cursor or arm of the other player. In the single-player condition subjects saw the same screen that would be displayed to two players in the game condition.

## Supporting Information

Text S1 Supplementary Materials
(0.15 MB PDF)
