# Supplementary material for: Nash Equilibria in Multi-Agent Motor Interactions
Source: PLoS Comput Biol. 2009 Aug 14;5(8):e1000468. doi: 10.1371/journal.pcbi.1000468 (PMC2714462; doi:10.1371/journal.pcbi.1000468)
Supplement: Text S1 — Supplementary Materials (0.15 MB PDF) [file pcbi.1000468.s001.pdf]

Supplementary Material

Nash equilibria in multi-agent motor interactions

D.A. Braun, P.A. Ortega and D.M. Wolpert

Contents

1 Experimental & Theoretical Setup **2**

2 The Bimanual Task **3**

3 The Two-Player Condition **5**

1 Experimental & Theoretical Setup

The most general theoretical framework for multi-agent motor interactions is *dynamic game theory* [1] in case of competing players and *optimal control theory* [4] in case of cooperating players. In our experiments the condition of cooperation and competition was translated into a one-player versus a two-player condition. In the one-player condition the two robot handles were controlled by the two arms of one person. In the two-player condition the two robot handles were controlled by two different players. In the one-player condition bimanual coordination can be understood by a player minimizing a cost functions that takes into account both task requirements and energy consumption (see [3] for reference). In the two-player condition both players try to minimize their cost respective functions but the global minimum must not be a stable solution. Dynamic game theory allows predicting the stable Nash solution in which players minimize their maximum loss.

In both optimal control theory and dynamic game theory analytic solutions to multi-agent control problems can be found if the problems can be formulated as *linear* dynamical systems. As described in the main text we conducted two experiments:

- The prisoner's dilemma motor game. Our version of the prisoner's dilemma defines a nonlinear dynamical system, because the force that is experienced by a player, depends on terms that are bilinear in the arm position of that player. This can be seen as follows: The force f depends both on the spring constant K and the y-position, such that $f = Ky$. The spring constant, however, depends on the x-positions of the two players such that $K(x_1, x_2) = \alpha x_1 + \beta x_2$. Thus, the force depends on terms bilinear in yx_1 and yx_2. To analyze the result of this experiment we therefore discretized the x_1x_2-space and performed a traditional discrete matrix game analysis (see [1] for reference).

- The rope-pulling game. This game can be expressed as a linear dynamical system. Each one of two players controls a robot handle, with position $\mathbf{x}_t^{H1}$ and $\mathbf{x}_t^{H2}$ in the horizontal plane respectively, which together control a virtual mass point such that

$$\mathbf{x}_t^C = \alpha \mathbb{D}_{\phi_1} \mathbf{x}_t^{H1} + \alpha \mathbb{D}_{\phi_2} \mathbf{x}_t^{H2} \tag{1}$$

  where $\mathbf{x}_t^C$ is the mass point position at time t and $\mathbb{D}_{\phi_1}$ and $\mathbb{D}_{\phi_2}$ are visuomotor rotation matrices and α is a scaling parameter. The scaling parameter was set to $\alpha = 2$ throughout our experiment to confine arm movements to a small space in order to avoid collision of the robot handles. Additionally, an isotropic spring was simulated on each robot handle to provide proprioceptive feedback of the distance moved. Since the spring was isotropic we do not need to model it explicitly and can use the moved distance as a proxy. In the experiment the visuomotor rotation matrices $\mathbb{D}_{\phi_i}$ were drawn randomly every 80 trials from the set $\{-135°, -90°, -45°, 0°, +45°\}$, but in the model we assume that the rotations are known to the player, which corresponds to a situation after learning has taken place.

In the following we present the analytical solution to the rope-pulling game.

2 The Bimanual Task

The optimal solution for the bimanual condition can be computed based on optimal feedback control for Linear-Quadratic-Gaussian (LQG) systems [4]. LQG models deal with linear dynamic systems, quadratic cost functions as performance criteria, and Gaussian random variables as noise. Here we consider the following model

$$\mathbf{x}_{t+1} = F\,\mathbf{x}_t + G\,\mathbf{u}_t + \xi_t \tag{2}$$

with the variables

$$\begin{array}{ll} \text{dynamic state} & \mathbf{x}_t \in \Re^n \\ \text{control signal} & \mathbf{u}_t \in \Re^m \\ \text{noise} & \xi_t \in \Re^n \end{array}$$

The random variable $\xi_t \in \Re^n$ is a realization of an independent, zero-mean, Gaussian noise process with covariance matrix $\mathbb{E}[\xi_{t_1}\xi_{t_2}^T] = \Omega_\xi\,\delta_{t_1 t_2}$. The system matrices F and G define the dynamics of the system. To simplify the analysis we consider an infinite horizon cost function

$$J = \frac{1}{2}\mathbb{E}\left[\sum_{t=0}^{\infty}\left\{\mathbf{x}_t^T\,Q\,\mathbf{x}_t + \mathbf{u}_t^T\,R\,\mathbf{u}_t\right\}\right] \tag{3}$$

with

$$\begin{array}{ll} \text{expected cumulative cost} & J \in \Re_+^* \\ \text{state cost matrix} & Q = Q^T \geq 0 \\ \text{control cost matrix} & R = R^T > 0 \end{array}$$

Time is discretized in bins of $10ms$. The model predictions can be seen in Figure S1.

Arm Model. Following previous studies [5, 6] the hands are modeled as point masses m with two-dimensional positions $\mathbf{p}^{H_i}(t)$ and velocities $\mathbf{v}^{H_i}(t) = \dot{\mathbf{p}}^{H_i}(t)$ with $i = 1, 2$. The two hands are designated by H_1 and H_2 respectively. The combined action of all muscles on the hands is represented by the force vectors $\mathbf{f}^{H_i}(t)$. The neural control signals $\mathbf{u}^{H_i}(t)$ are transformed to these forces through second-order muscle-like low-pass filters with time constants τ_1 and τ_2. In every instant of time, the two hand motions are mapped to the virtual cursor motion by equation (1). Thus, the virtual cursor position $\mathbf{p}(t)$ is related to the hand positions by $\mathbf{p}(t) = \mathbb{D}_{\phi_1}\,\mathbf{p}^{H_1}(t) + \mathbb{D}_{\phi_2}\,\mathbf{p}^{H_2}(t)$. Put together, this yields the following system equations

$$\ddot{\mathbf{p}}(t) = \frac{1}{m}\sum_{i=1}^{2}\mathbb{D}_{\phi_i}\,\mathbf{f}^{H_i}(t) \tag{4}$$

$$\tau_1\,\tau_2\,\ddot{\mathbf{f}}^{H_i}(t) + (\tau_1+\tau_2)\dot{\mathbf{f}}^{H_i}(t) + \mathbf{f}^{H_i}(t) = \mathbf{u}^{H_i}(t) \tag{5}$$

Equation (5) can be written equivalently as a pair of coupled first-order filters with outputs $\mathbf{g}$ and $\mathbf{f}$. This allows to formulate the state space vector $\mathbf{x}(t) \in \Re^{16}$ as

$$\mathbf{x}(t) = \begin{bmatrix} p^x(t) & v^{x,H_1}(t) & f^{x,H_1}(t) & g^{x,H_1}(t) & v^{x,H_2}(t) & f^{x,H_2}(t) & g^{x,H_2}(t) & p^{\text{TARGET},x} & \cdots \\ \cdots & p^y(t) & v^{y,H_1}(t) & f^{y,H_1}(t) & g^{y,H_1}(t) & v^{y,H_2}(t) & f^{y,H_2}(t) & g^{y,H_2}(t) & p^{\text{TARGET},y} \end{bmatrix}^T$$

where the target location is absorbed in the state vector. When discretizing the above equations with time bin Δ the following system matrices are obtained

$$F = \begin{pmatrix}
1 & \Delta\cos(\phi_1) & 0 & 0 & \Delta\cos(\phi_2) & 0 & 0 & 0 & 0 & -\Delta\sin(\phi_1) & 0 & 0 & -\Delta\sin(\phi_2) & 0 & 0 & 0 \\
0 & 1 & \frac{\Delta}{m} & 0 & 0 & 0 & 0 & 0 & 0 & 0 & 0 & 0 & 0 & 0 & 0 & 0 \\
0 & 0 & 1-\frac{\Delta}{\tau_2} & \frac{\Delta}{\tau_2} & 0 & 0 & 0 & 0 & 0 & 0 & 0 & 0 & 0 & 0 & 0 & 0 \\
0 & 0 & 0 & 1-\frac{\Delta}{\tau_1} & 0 & 0 & 0 & 0 & 0 & 0 & 0 & 0 & 0 & 0 & 0 & 0 \\
0 & 0 & 0 & 0 & 1 & \frac{\Delta}{m} & 0 & 0 & 0 & 0 & 0 & 0 & 0 & 0 & 0 & 0 \\
0 & 0 & 0 & 0 & 0 & 1-\frac{\Delta}{\tau_2} & \frac{\Delta}{\tau_2} & 0 & 0 & 0 & 0 & 0 & 0 & 0 & 0 & 0 \\
0 & 0 & 0 & 0 & 0 & 0 & 1-\frac{\Delta}{\tau_1} & 0 & 0 & 0 & 0 & 0 & 0 & 0 & 0 & 0 \\
0 & 0 & 0 & 0 & 0 & 0 & 0 & 1 & 0 & 0 & 0 & 0 & 0 & 0 & 0 & 0 \\
0 & \Delta\sin(\phi_1) & 0 & 0 & \Delta\sin(\phi_2) & 0 & 0 & 0 & 1 & \Delta\cos(\phi_1) & 0 & 0 & \Delta\cos(\phi_2) & 0 & 0 & 0 \\
0 & 0 & 0 & 0 & 0 & 0 & 0 & 0 & 0 & 1 & \frac{\Delta}{m} & 0 & 0 & 0 & 0 & 0 \\
0 & 0 & 0 & 0 & 0 & 0 & 0 & 0 & 0 & 0 & 1-\frac{\Delta}{\tau_2} & \frac{\Delta}{\tau_2} & 0 & 0 & 0 & 0 \\
0 & 0 & 0 & 0 & 0 & 0 & 0 & 0 & 0 & 0 & 0 & 1-\frac{\Delta}{\tau_1} & 0 & 0 & 0 & 0 \\
0 & 0 & 0 & 0 & 0 & 0 & 0 & 0 & 0 & 0 & 0 & 0 & 1 & \frac{\Delta}{m} & 0 & 0 \\
0 & 0 & 0 & 0 & 0 & 0 & 0 & 0 & 0 & 0 & 0 & 0 & 0 & 1-\frac{\Delta}{\tau_2} & \frac{\Delta}{\tau_2} & 0 \\
0 & 0 & 0 & 0 & 0 & 0 & 0 & 0 & 0 & 0 & 0 & 0 & 0 & 0 & 1-\frac{\Delta}{\tau_1} & 0 \\
0 & 0 & 0 & 0 & 0 & 0 & 0 & 0 & 0 & 0 & 0 & 0 & 0 & 0 & 0 & 1
\end{pmatrix}$$

$$G = \begin{pmatrix}
0 & 0 & 0 & 0 \\
0 & 0 & 0 & 0 \\
0 & 0 & 0 & 0 \\
\frac{\Delta}{\tau_1} & 0 & 0 & 0 \\
0 & 0 & 0 & 0 \\
0 & 0 & 0 & 0 \\
0 & 0 & \frac{\Delta}{\tau_1} & 0 \\
0 & 0 & 0 & 0 \\
0 & 0 & 0 & 0 \\
0 & 0 & 0 & 0 \\
0 & 0 & 0 & 0 \\
0 & \frac{\Delta}{\tau_1} & 0 & 0 \\
0 & 0 & 0 & 0 \\
0 & 0 & 0 & 0 \\
0 & 0 & 0 & \frac{\Delta}{\tau_1} \\
0 & 0 & 0 & 0
\end{pmatrix}$$

In our simulations we set $m = 1kg$ and $\tau_1 = \tau_2 = 0.04s$.

Cost Matrices. The cost matrices Q and R are defined as follows:

$$Q = \begin{pmatrix}
w_p & 0 & 0 & 0 & 0 & 0 & 0 & -w_p & 0 & 0 & 0 & 0 & 0 & 0 & 0 & 0 \\
0 & w_v & 0 & 0 & 0 & 0 & 0 & 0 & 0 & 0 & 0 & 0 & 0 & 0 & 0 & 0 \\
0 & 0 & 0 & 0 & 0 & 0 & 0 & 0 & 0 & 0 & 0 & 0 & 0 & 0 & 0 & 0 \\
0 & 0 & 0 & 0 & 0 & 0 & 0 & 0 & 0 & 0 & 0 & 0 & 0 & 0 & 0 & 0 \\
0 & 0 & 0 & 0 & w_v & 0 & 0 & 0 & 0 & 0 & 0 & 0 & 0 & 0 & 0 & 0 \\
0 & 0 & 0 & 0 & 0 & 0 & 0 & 0 & 0 & 0 & 0 & 0 & 0 & 0 & 0 & 0 \\
0 & 0 & 0 & 0 & 0 & 0 & 0 & 0 & 0 & 0 & 0 & 0 & 0 & 0 & 0 & 0 \\
-w_p & 0 & 0 & 0 & 0 & 0 & 0 & w_p & 0 & 0 & 0 & 0 & 0 & 0 & 0 & 0 \\
0 & 0 & 0 & 0 & 0 & 0 & 0 & 0 & w_p & 0 & 0 & 0 & 0 & 0 & 0 & -w_p \\
0 & 0 & 0 & 0 & 0 & 0 & 0 & 0 & 0 & w_v & 0 & 0 & 0 & 0 & 0 & 0 \\
0 & 0 & 0 & 0 & 0 & 0 & 0 & 0 & 0 & 0 & 0 & 0 & 0 & 0 & 0 & 0 \\
0 & 0 & 0 & 0 & 0 & 0 & 0 & 0 & 0 & 0 & 0 & 0 & 0 & 0 & 0 & 0 \\
0 & 0 & 0 & 0 & 0 & 0 & 0 & 0 & 0 & 0 & 0 & 0 & w_v & 0 & 0 & 0 \\
0 & 0 & 0 & 0 & 0 & 0 & 0 & 0 & 0 & 0 & 0 & 0 & 0 & 0 & 0 & 0 \\
0 & 0 & 0 & 0 & 0 & 0 & 0 & 0 & 0 & 0 & 0 & 0 & 0 & 0 & 0 & 0 \\
0 & 0 & 0 & 0 & 0 & 0 & 0 & 0 & -w_p & 0 & 0 & 0 & 0 & 0 & 0 & w_p
\end{pmatrix}$$

$$R = w_e \begin{pmatrix}
1 & 0 & 0 & 0 \\
0 & 1 & 0 & 0 \\
0 & 0 & 1 & 0 \\
0 & 0 & 0 & 1
\end{pmatrix}$$

Importantly, the Q-matrix punishes deviations from the x- and y-components of the virtual target equally, i.e. both components must be optimized as required for global optimality. In our simulations we set $w_p = 1$, $w_v = 0.02$ and $w_e = 10^{-5}$.

Optimal Policy. The optimal control policy is given by

$$\mathbf{u}_t = -L\,\mathbf{x}_t \tag{6}$$

with

$$L = \left(R + G^T\, S\, G\right)^{-1} G^T\, S\, F \tag{7}$$

The matrix S can be easily computed by solving the algebraic Riccati equation

$$S = Q + F^T\, S\, F - F^T\, S\, G\, \left(R + G^T\, S\, G\right)^{-1} G^T\, S\, F \tag{8}$$

The optimal policy $\mathbf{u}_t$ is 4-dimensional and comprises the optimal control policies for both hands $\mathbf{u}_t = [\mathbf{u}_t^{H1}; \mathbf{u}_t^{H2}]$.

3 The Two-Player Condition

Nash strategies can be defined for linear quadratic games [1]. A linear quadratic game is characterized by linear system equations and quadratic cost functions for all players. In the following we assume

$$\mathbf{x}_{t+1} = F\, \mathbf{x}_t + G_1\, \mathbf{u}_t^{P1} + G_2\, \mathbf{u}_t^{P2} \tag{9}$$

with the variables

dynamic state	$\mathbf{x}_t \in \Re^n$
control signal player 1	$\mathbf{u}_t^{P1} \in \Re^m$
control signal player 2	$\mathbf{u}_t^{P2} \in \Re^m$

The system matrices F, G_1 and G_2 define the dynamics of the system. In this two-player game there are two cost functions to consider

$$J_1 = \frac{1}{2}\mathbb{E}\left[\sum_{t=0}^{\infty} \left\{\mathbf{x}_t^T\, Q_1\, \mathbf{x}_t + \left(\mathbf{u}_t^{P1}\right)^T R_1\, \mathbf{u}_t^{P1}\right\}\right] \tag{10}$$

$$J_2 = \frac{1}{2}\mathbb{E}\left[\sum_{t=0}^{\infty} \left\{\mathbf{x}_t^T\, Q_2\, \mathbf{x}_t + \left(\mathbf{u}_t^{P2}\right)^T R_2\, \mathbf{u}_t^{P2}\right\}\right] \tag{11}$$

with

expected cumulative cost player 1	$J_1 \in \Re_+^*$
expected cumulative cost player 2	$J_2 \in \Re_+^*$
state cost matrix player 1	$Q_1 = Q_1^T \geq 0$
state cost matrix player 2	$Q_2 = Q_2^T \geq 0$
control cost matrix player 1	$R_1 = R_1^T > 0$
control cost matrix player 2	$R_2 = R_2^T > 0$

In the following we assume $R_1 = R_2$, i.e. no differences in the penalization of efforts between the two players. The model predictions can be seen in Figure S2.

Arm Model. The system dynamics are identical to the system dynamics in the bimanual condition. Note the equivalence between $G = [G_1\; G_2]$.

Cost Matrices. The cost matrices for the two players are:

$$Q_1 = \begin{pmatrix}
w_p & 0 & 0 & 0 & 0 & 0 & 0 & -w_p & 0 & 0 & 0 & 0 & 0 & 0 & 0 & 0 \\
0 & w_v & 0 & 0 & 0 & 0 & 0 & 0 & 0 & 0 & 0 & 0 & 0 & 0 & 0 & 0 \\
0 & 0 & 0 & 0 & 0 & 0 & 0 & 0 & 0 & 0 & 0 & 0 & 0 & 0 & 0 & 0 \\
0 & 0 & 0 & 0 & 0 & 0 & 0 & 0 & 0 & 0 & 0 & 0 & 0 & 0 & 0 & 0 \\
0 & 0 & 0 & 0 & w_v & 0 & 0 & 0 & 0 & 0 & 0 & 0 & 0 & 0 & 0 & 0 \\
0 & 0 & 0 & 0 & 0 & 0 & 0 & 0 & 0 & 0 & 0 & 0 & 0 & 0 & 0 & 0 \\
0 & 0 & 0 & 0 & 0 & 0 & 0 & 0 & 0 & 0 & 0 & 0 & 0 & 0 & 0 & 0 \\
-w_p & 0 & 0 & 0 & 0 & 0 & 0 & w_p & 0 & 0 & 0 & 0 & 0 & 0 & 0 & 0 \\
0 & 0 & 0 & 0 & 0 & 0 & 0 & 0 & 0 & 0 & 0 & 0 & 0 & 0 & 0 & 0 \\
0 & 0 & 0 & 0 & 0 & 0 & 0 & 0 & 0 & 0 & 0 & 0 & 0 & 0 & 0 & 0 \\
0 & 0 & 0 & 0 & 0 & 0 & 0 & 0 & 0 & 0 & 0 & 0 & 0 & 0 & 0 & 0 \\
0 & 0 & 0 & 0 & 0 & 0 & 0 & 0 & 0 & 0 & 0 & 0 & 0 & 0 & 0 & 0 \\
0 & 0 & 0 & 0 & 0 & 0 & 0 & 0 & 0 & 0 & 0 & 0 & 0 & 0 & 0 & 0 \\
0 & 0 & 0 & 0 & 0 & 0 & 0 & 0 & 0 & 0 & 0 & 0 & 0 & 0 & 0 & 0 \\
0 & 0 & 0 & 0 & 0 & 0 & 0 & 0 & 0 & 0 & 0 & 0 & 0 & 0 & 0 & 0 \\
0 & 0 & 0 & 0 & 0 & 0 & 0 & 0 & 0 & 0 & 0 & 0 & 0 & 0 & 0 & 0
\end{pmatrix}$$

$$Q_2 = \begin{pmatrix}
0 & 0 & 0 & 0 & 0 & 0 & 0 & 0 & 0 & 0 & 0 & 0 & 0 & 0 & 0 & 0 \\
0 & 0 & 0 & 0 & 0 & 0 & 0 & 0 & 0 & 0 & 0 & 0 & 0 & 0 & 0 & 0 \\
0 & 0 & 0 & 0 & 0 & 0 & 0 & 0 & 0 & 0 & 0 & 0 & 0 & 0 & 0 & 0 \\
0 & 0 & 0 & 0 & 0 & 0 & 0 & 0 & 0 & 0 & 0 & 0 & 0 & 0 & 0 & 0 \\
0 & 0 & 0 & 0 & 0 & 0 & 0 & 0 & 0 & 0 & 0 & 0 & 0 & 0 & 0 & 0 \\
0 & 0 & 0 & 0 & 0 & 0 & 0 & 0 & 0 & 0 & 0 & 0 & 0 & 0 & 0 & 0 \\
0 & 0 & 0 & 0 & 0 & 0 & 0 & 0 & 0 & 0 & 0 & 0 & 0 & 0 & 0 & 0 \\
0 & 0 & 0 & 0 & 0 & 0 & 0 & 0 & 0 & 0 & 0 & 0 & 0 & 0 & 0 & 0 \\
0 & 0 & 0 & 0 & 0 & 0 & 0 & 0 & w_p & 0 & 0 & 0 & 0 & 0 & 0 & -w_p \\
0 & 0 & 0 & 0 & 0 & 0 & 0 & 0 & 0 & w_v & 0 & 0 & 0 & 0 & 0 & 0 \\
0 & 0 & 0 & 0 & 0 & 0 & 0 & 0 & 0 & 0 & 0 & 0 & 0 & 0 & 0 & 0 \\
0 & 0 & 0 & 0 & 0 & 0 & 0 & 0 & 0 & 0 & 0 & 0 & 0 & 0 & 0 & 0 \\
0 & 0 & 0 & 0 & 0 & 0 & 0 & 0 & 0 & 0 & 0 & 0 & w_v & 0 & 0 & 0 \\
0 & 0 & 0 & 0 & 0 & 0 & 0 & 0 & 0 & 0 & 0 & 0 & 0 & 0 & 0 & 0 \\
0 & 0 & 0 & 0 & 0 & 0 & 0 & 0 & 0 & 0 & 0 & 0 & 0 & 0 & 0 & 0 \\
0 & 0 & 0 & 0 & 0 & 0 & 0 & 0 & -w_p & 0 & 0 & 0 & 0 & 0 & 0 & w_p
\end{pmatrix}$$

$$R_1 = R_2 = w_e \begin{pmatrix} 1 & 0 \\ 0 & 1 \end{pmatrix}$$

Importantly, in the game each player only optimizes his component of the projected virtual cursor independently. This is reflected in the Q-matrices. Note that there is a relation between the Q-matrix in the bimanual condition and the Q_i-matrices in the game condition, namely $Q = Q_1 + Q_2$. The parameters w_p, w_v and w_e were set equally in both conditions.

Nash Policy. The Nash equilibrium policies can be computed to be

$$\mathbf{u}_t^{P1} = -L_1\,\mathbf{x}_t \tag{12}$$

$$\mathbf{u}_t^{P2} = -L_2\,\mathbf{x}_t \tag{13}$$

with

$$L_i = \left(R + G_i^T\,S_i\,G_i\right)^{-1} G_i^T\,S_i\,F \tag{14}$$

The matrices S_i can be computed by solving the coupled algebraic Riccati equations

$$S_1 = Q_1 + F^T S_1 F - F^T S_1 G_1 \left(R_1 + G_1^T S_1 G_1\right) G_1^T S_1 F - F^T S_1 G_2 \left(R_2 + G_2^T S_2 G_2\right) G_2^T S_2 F - F^T S_2 G_2 \left(R_2 + G_2^T S_2 G_2\right) G_2^T S_1 F \tag{15}$$

$$S_2 = Q_2 + F^T S_2 F - F^T S_2 G_2 \left(R_2 + G_2^T S_2 G_2\right) G_2^T S_2 F - F^T S_2 G_1 \left(R_1 + G_1^T S_1 G_1\right) G_1^T S_1 F - F^T S_1 G_1 \left(R_1 + G_1^T S_1 G_1\right) G_1^T S_2 F \tag{16}$$

The Nash policies $\mathbf{u}_t^{P1}$ and $\mathbf{u}_t^{P2}$ give the optimal control policies for both players in equilibrium.

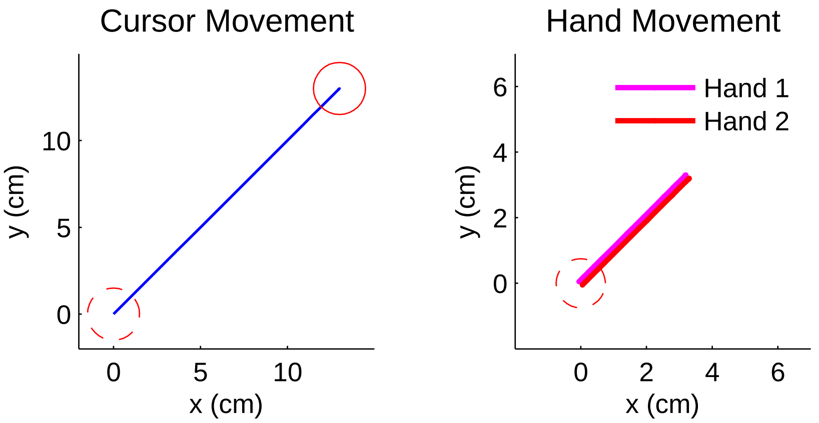

Figure S1. Predictions for cursor and hand trajectories in our version of the rope pulling game for cooperating players. In this example the rotation matrices for each hand have been set to the identity matrix, the gain has been set to $\alpha = 2$. The hand trajectories are shown slightly displaced to allow visibility. The optimal feedback control model allows predicting the direction of the hand movements for arbitrary settings of the rotation matrices.

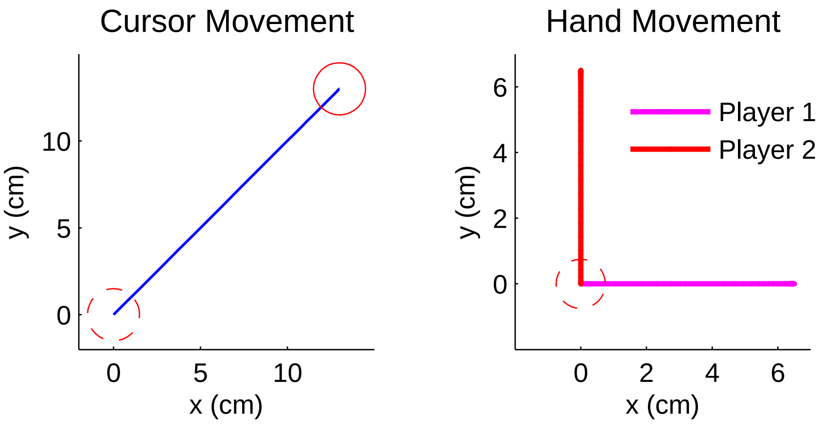

Figure S2. Predictions for cursor and hand trajectories in our version of the rope pulling game for competitive players. In this example the rotation matrices for each player have been set to the identity matrix, the gain has been set to $\alpha = 2$. The dynamic game control model allows predicting the direction of the hand movements of each player for arbitrary settings of the rotation matrices.

References

[1] Basar T, Olsder GJ (1987) Dynamic noncooperative game theory. Society for Industrial Mathematics

[2] Bays PM, Wolpert DM (2006) Actions and consequences in bimanual interaction are represented in different coordinate systems. J Neurosci 26: 7121-7126

[3] Diedrichsen J (2007) Optimal task-dependent changes of bimanual feedback control and adaptation. Curr. Biol. 17: 16751679

[4] Stengel RF (1994) Optimal control and estimation, Dover Publications

[5] Todorov E, Jordan MI (2002) Optimal feedback control as a theory of motor coordination, Nat Neurosci. 5:1226-1235

[6] Winter DA (1990) Biomechanics and motor control of human movement, John Wiley and Sons, Inc., New York
